# Supplementary material for: Biologically Oriented Hybrids of Indole and Hydantoin Derivatives
Source: Molecules. 2023 Jan 6;28(2):602. doi: 10.3390/molecules28020602 (PMC9866919; doi:10.3390/molecules28020602)
Supplement: Supplementary file 1 [file molecules-28-00602-s001.zip › molecules-2115192-supplementary.pdf]

Electronic supplementary information

Biologically Oriented Hybrids of Indole and Hydantoin Derivatives

Konstantin A. Kochetkov *, Olga N. Gorunova and Natalia A. Bystrova

A. N. Nesmeyanov Institute of Organoelement Compounds, Russian Academy of Sciences, 28 ul. Vavilova, 119991 Moscow, Russia

***** *Correspondence: const@ineos.ac.ru; Tel.: +7-499-135-5085*

Content

I. Preparation of substances and seeds for testing.

II. Methodology of the experiment (Formulas S1-S3).

III. Figure S1. The development of the root system of wheat seeds on the second day.

IV. Figure S2. The development of the root system and shoots of wheat on the seventh day.

V. References.

VI. ^{1}H, ^{13}C NMR and COSY, gHSQC, gHMBC spectra of hybrids **3** (Figures **S3-S23**).

1. Preparation of substances and seeds for testing

Aqueous solutions with concentrations of 0.4×10^{-6}M or 0.4 µM were prepared for all compounds. Wheat seeds (*Triticum aestivum* L.) of the "Darya ® " variety, crop 2020, provided by LLC "Zhito", Oktyabrsky district, Ryazan, Ryazan region, Russia 54.609836° S.w., 39.80188° V.D. were used. These seeds are registered in the State Register of Breeding Achievements of the Russian Federation No. 9705798. Before starting work, wheat seeds *Triticum aestivum* L. sterilized with 0.2% sodium hydrochloride solution for 10 minutes, washed three times with distilled water, dried at a temperature of 30 ° C for 48 hours. The dried seeds were stored at a temperature of 5 ^{o}C.

2. Methodology of the experiment

Four independent series of experiments in identical cameras with phyto-LED UFO lighting-79-01-00 with a wavelength of Red 615/ Blu 457 nm with an intensity of at least 250 lux were carried out. The illumination of the samples is 12/12 hours. The relative humidity of the air was 50 ± 2%. The temperature is 20 ± 2°C. The duration of the experiment is 7 days.

Fifty (50) pieces of dry sterilized seeds were placed on filter paper in rectangular Petri dishes 75x85 (mm), treated by spraying with the studied compounds. Wheat grains were treated with 0.335±0.003 ml of compound solutions.

Vertical spraying was carried out in an isolated box with disposable screen. After spraying, the screen was removed. The surface of the box was disinfected and created with paper napkins. After spraying, the seeds were covered with filter paper and 10 ml of distilled water was poured. Then the Petri dishes with a lid were moved to the growth chamber. The first 24 hours of the experiment were conducted in the dark. The seeds were aired every day. Petri dishes were opened for 25 minutes, and 5-10 ml of distilled water was added so that the seeds did not dry out. On the third day of the experiment, the lids of the Petri dishes were removed so that the shoots grew.

The germination potential of wheat seeds was determined 24 hours after the start of the experiment according to **Formula 1:**

Germination potential (%) = [Number of germination seeds 1 d / Number of total seed] x 100 (1)

Seed germination was calculated by **Formula 2** after the end of the experiment:

Germination (%) = [Number of germination seeds 7 d / Number of total seed] x 100 (2)

Fifteen shoots were randomly selected from Petri dishes to determine the length of the roots and the height of the shoots, as well as the relative water content (RWC). The last watering of the shoots was carried out 96 hours after the start of the experiment (the fourth day of the experiment).

RWC was determined within three days after the end of the experiment according to **Formula 3**:

$$\textbf{RWC \% = [(TW (FW - DW) / - DW)]} \times \textbf{100} \quad (\textbf{3})$$

where: FW = fresh weight; TW = obese weight; DW = dry weight.

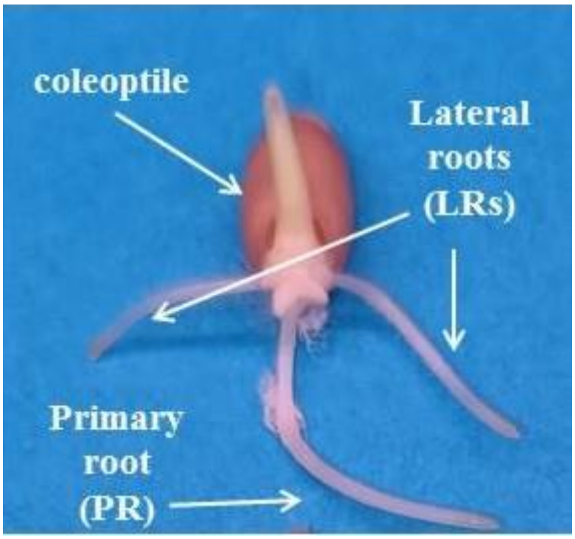

Figure S1. The development of the root system of wheat seeds on the second day.

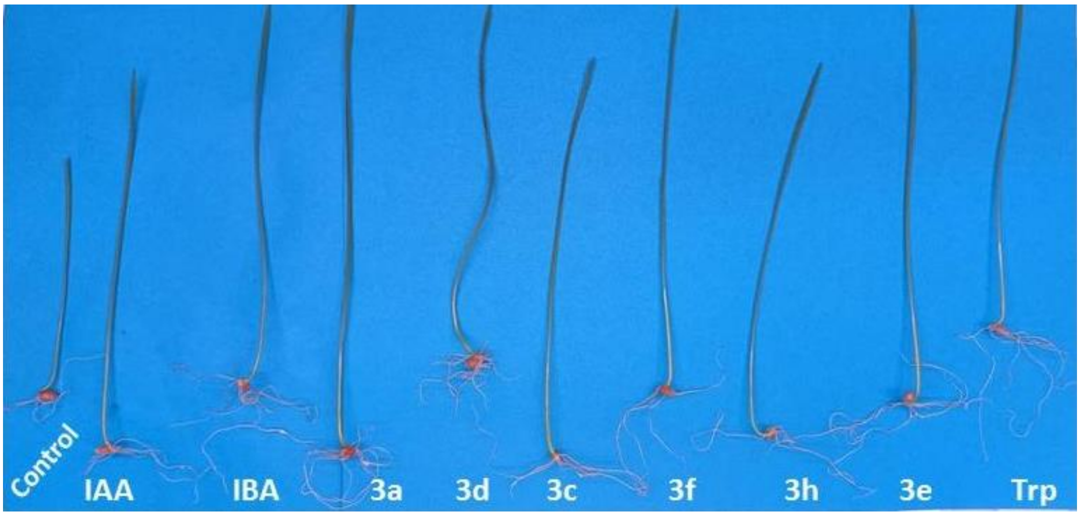

Figure S2. The development of the root system and shoots of wheat on the seventh day.

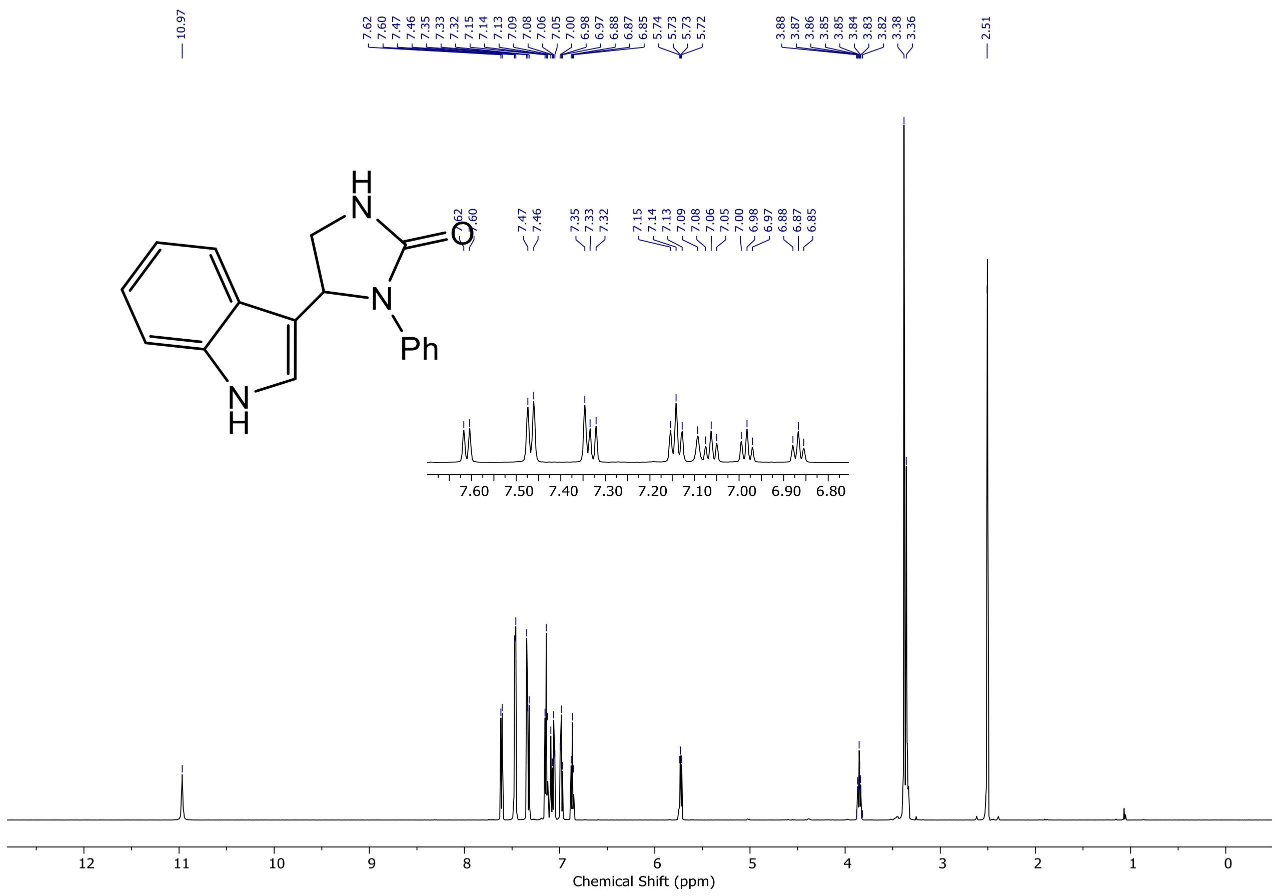

Figure S3. ^{1}H NMR spectrum of 5-(1*H*-Indol-3-yl)-1-phenylimidazolidin-2-one **3a**.

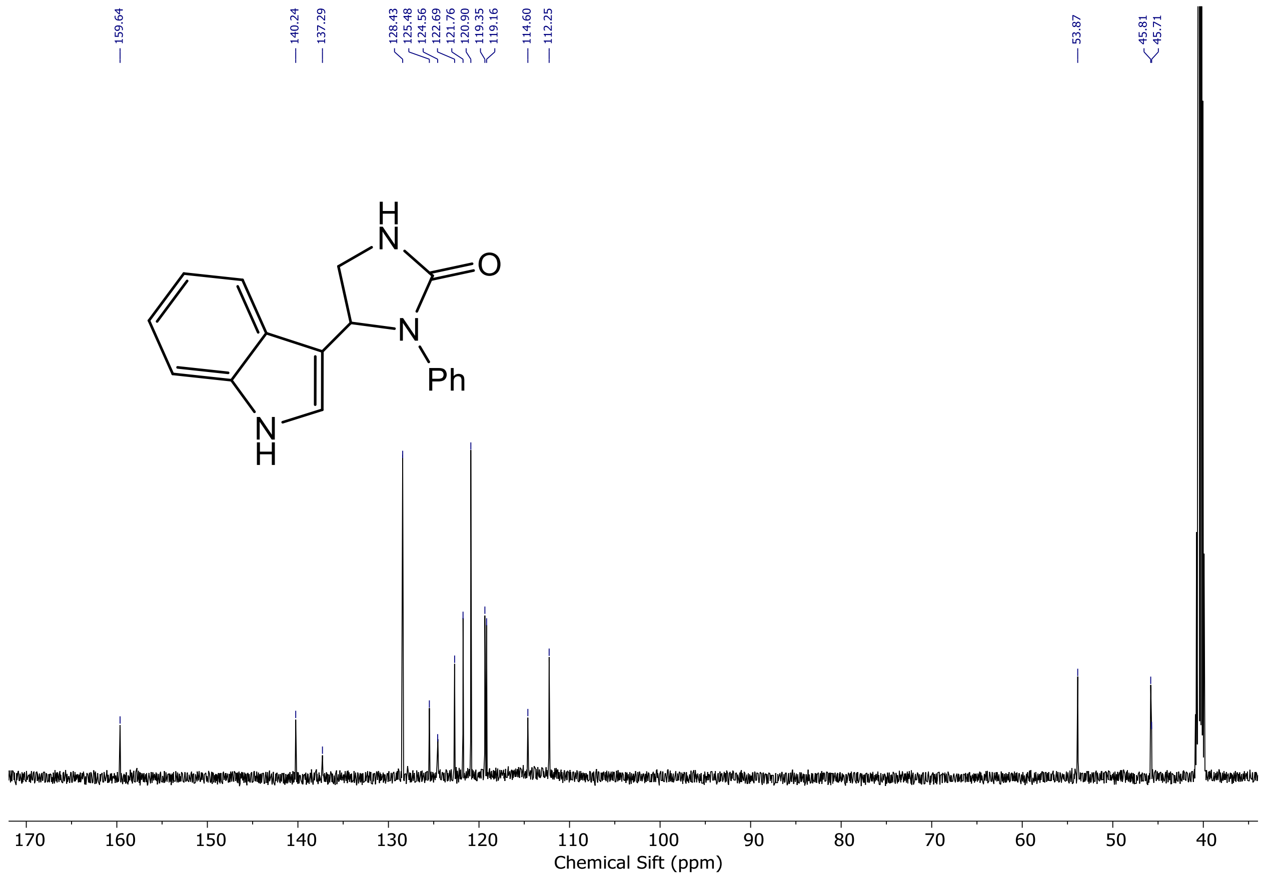

Figure S4. ^{13}H NMR spectrum of 5-(1*H*-Indol-3-yl)-1-phenylimidazolidin-2-one **3a**

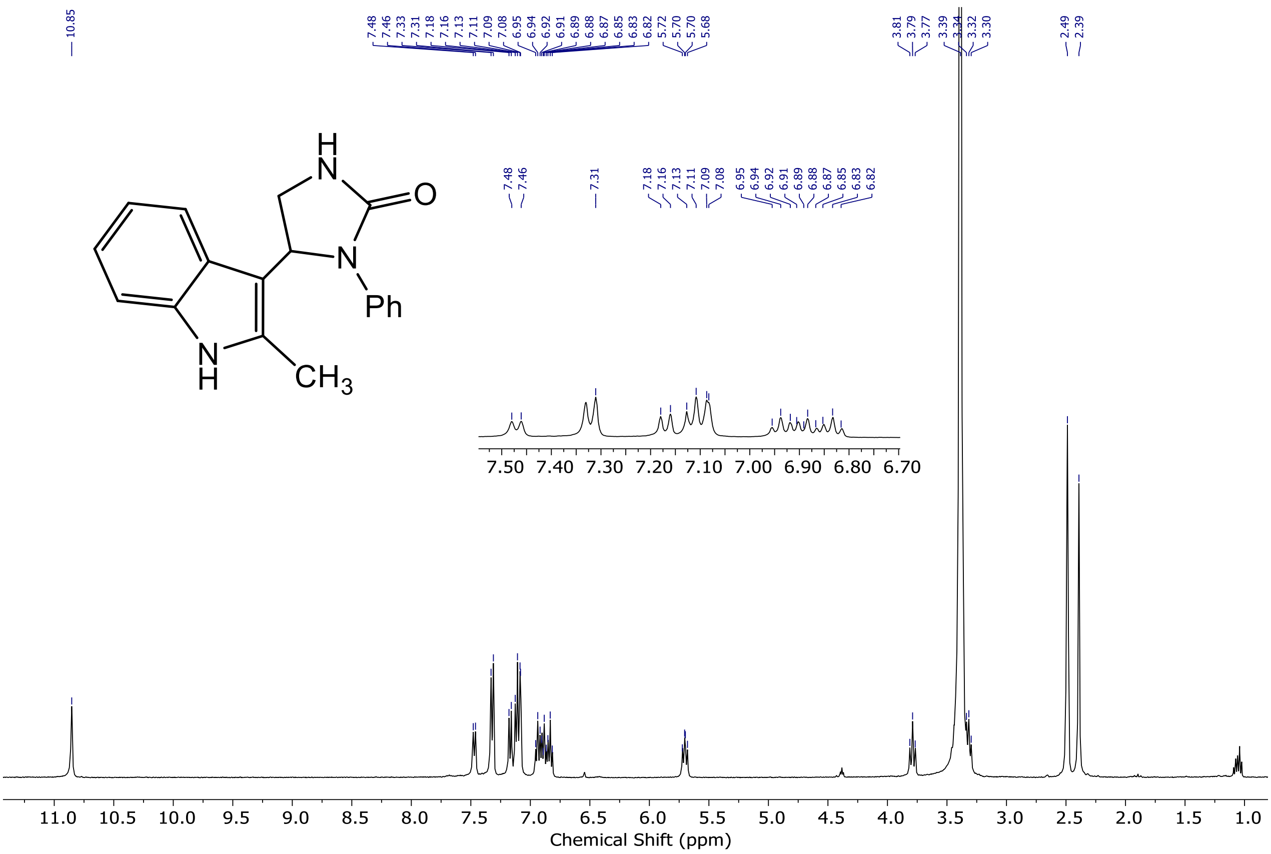

Figure S5. ^{1}H NMR spectrum of 5-(2-methyl-1*H*-indol-3-yl)-1-phenylimidazolidin-2-one **3b**.

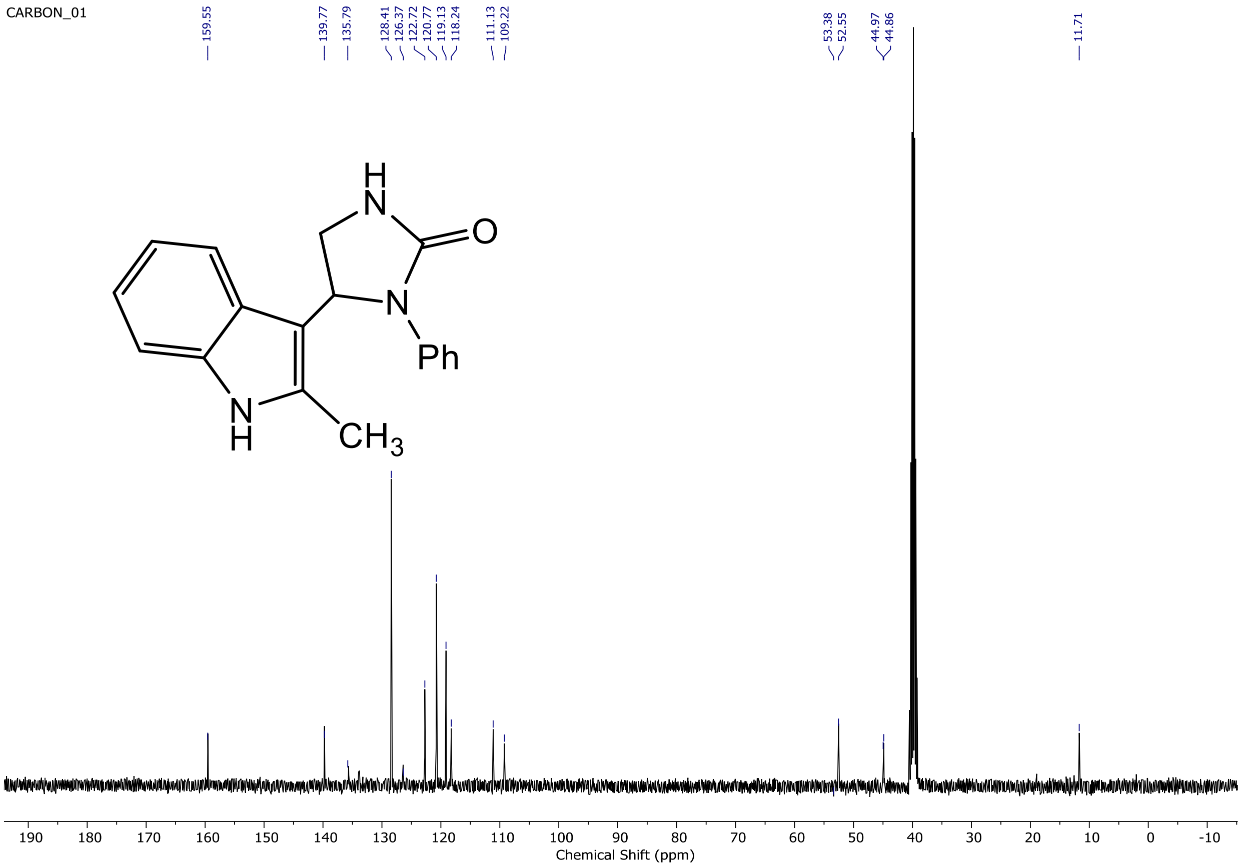

Figure S6. ^{13}C NMR spectrum of 5-(2-methyl-1*H*-indol-3-yl)-1-phenylimidazolidin-2-one **3b**.

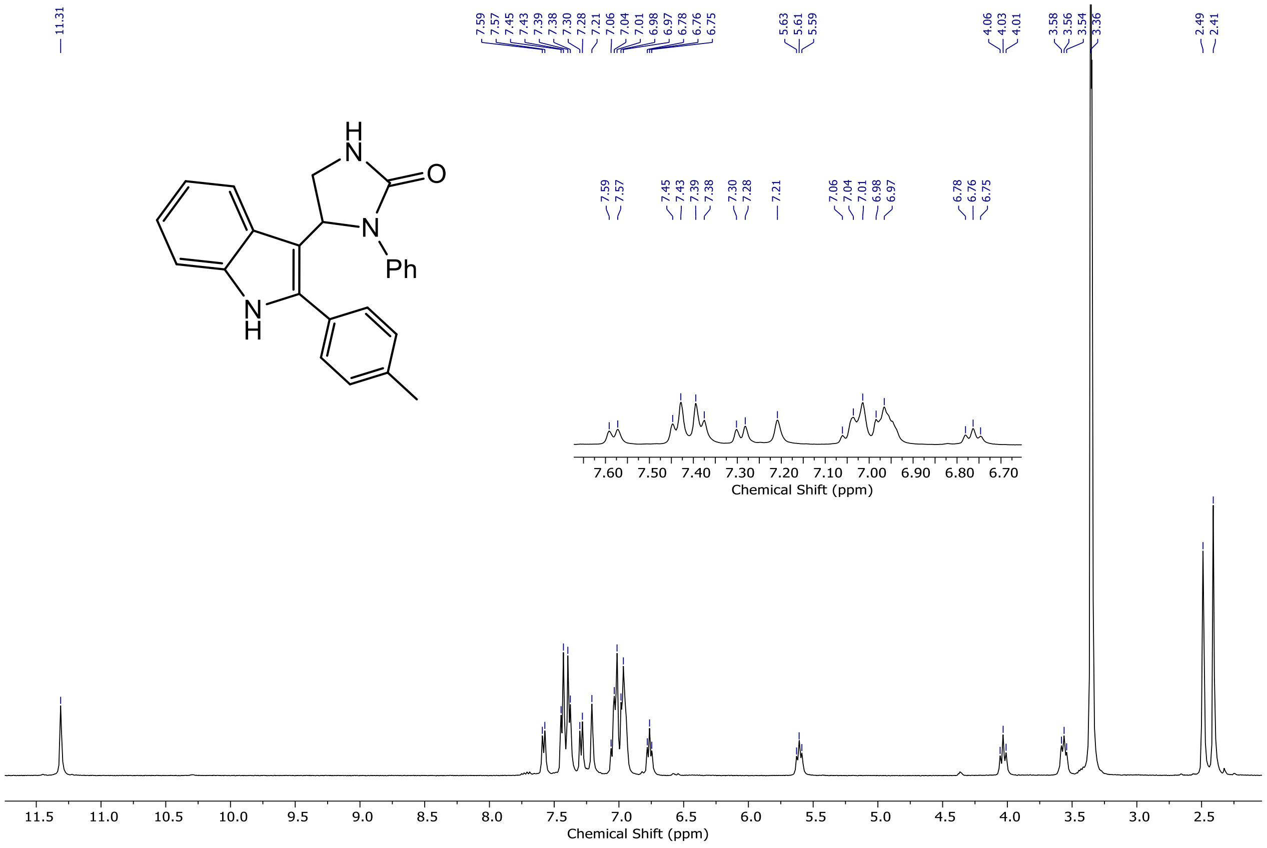

Figure S7. ^{1}H NMR spectrum of 5-(2-*p*-tolyl-1*H*-indol-3-yl)-1-phenylimidazolidin-2-one **3c**.

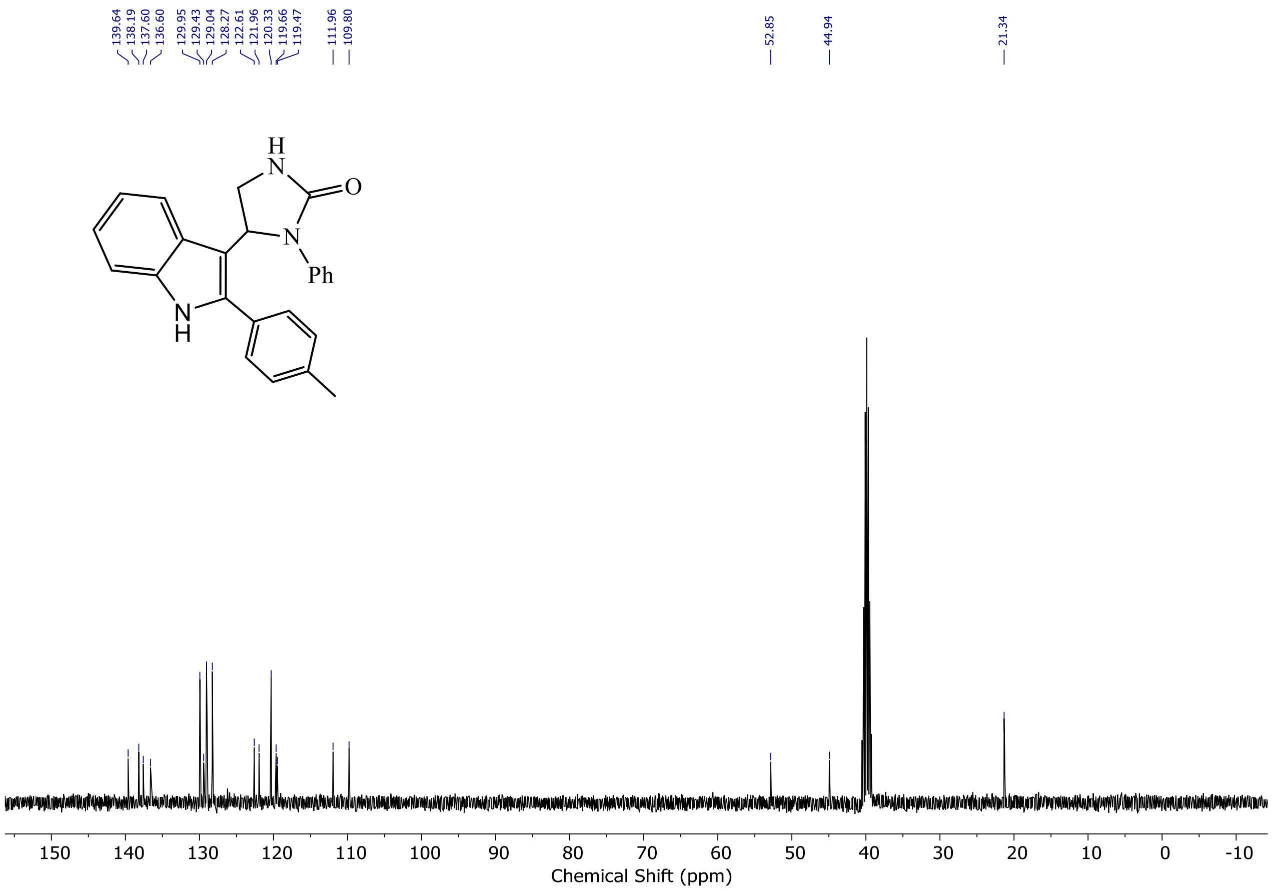

Figure S8. ^{13}C NMR spectrum of 5-(2-*p*-tolyl-1*H*-indol-3-yl)-1-phenylimidazolidin-2-one **3c.**

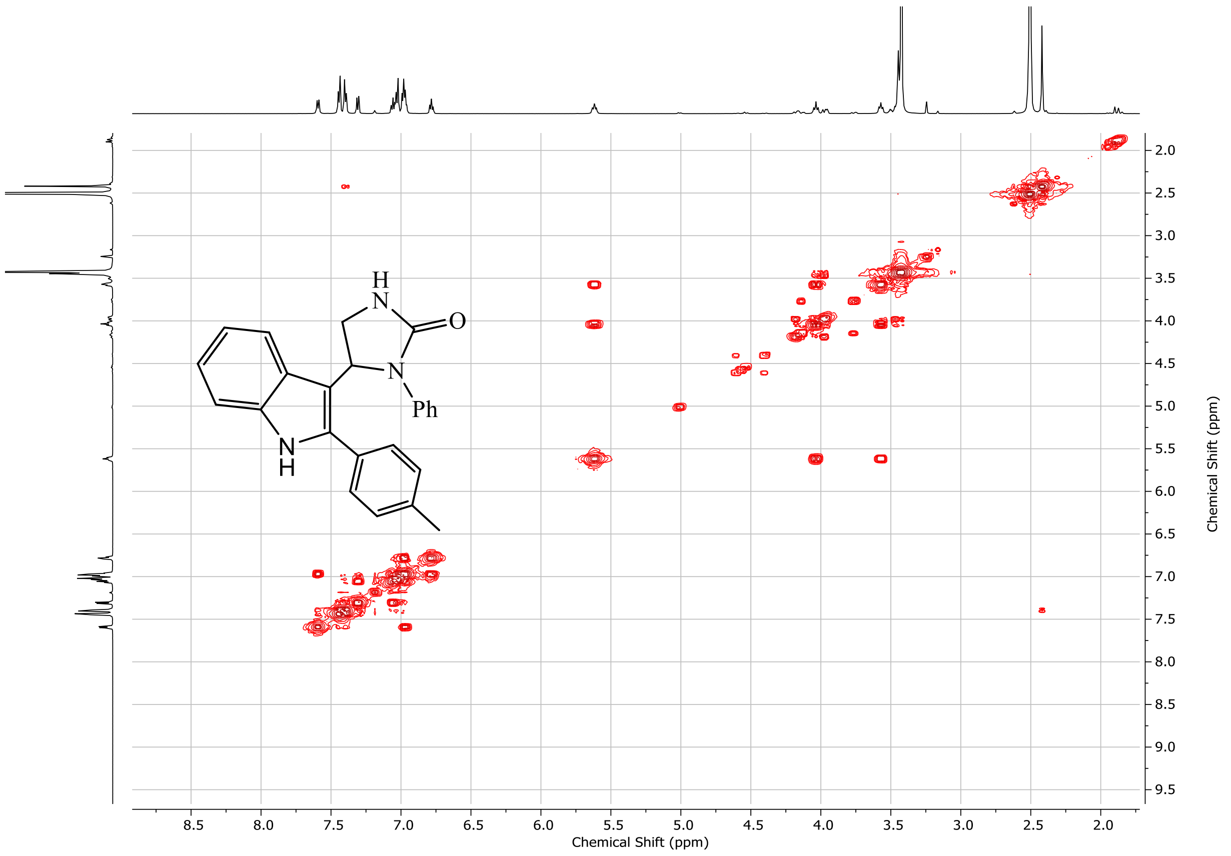

Figure S9. gCOSY NMR spectrum of 5-(2-*p*-tolyl-1*H*-indol-3-yl)-1-phenylimidazolidin-2-one **3c**.

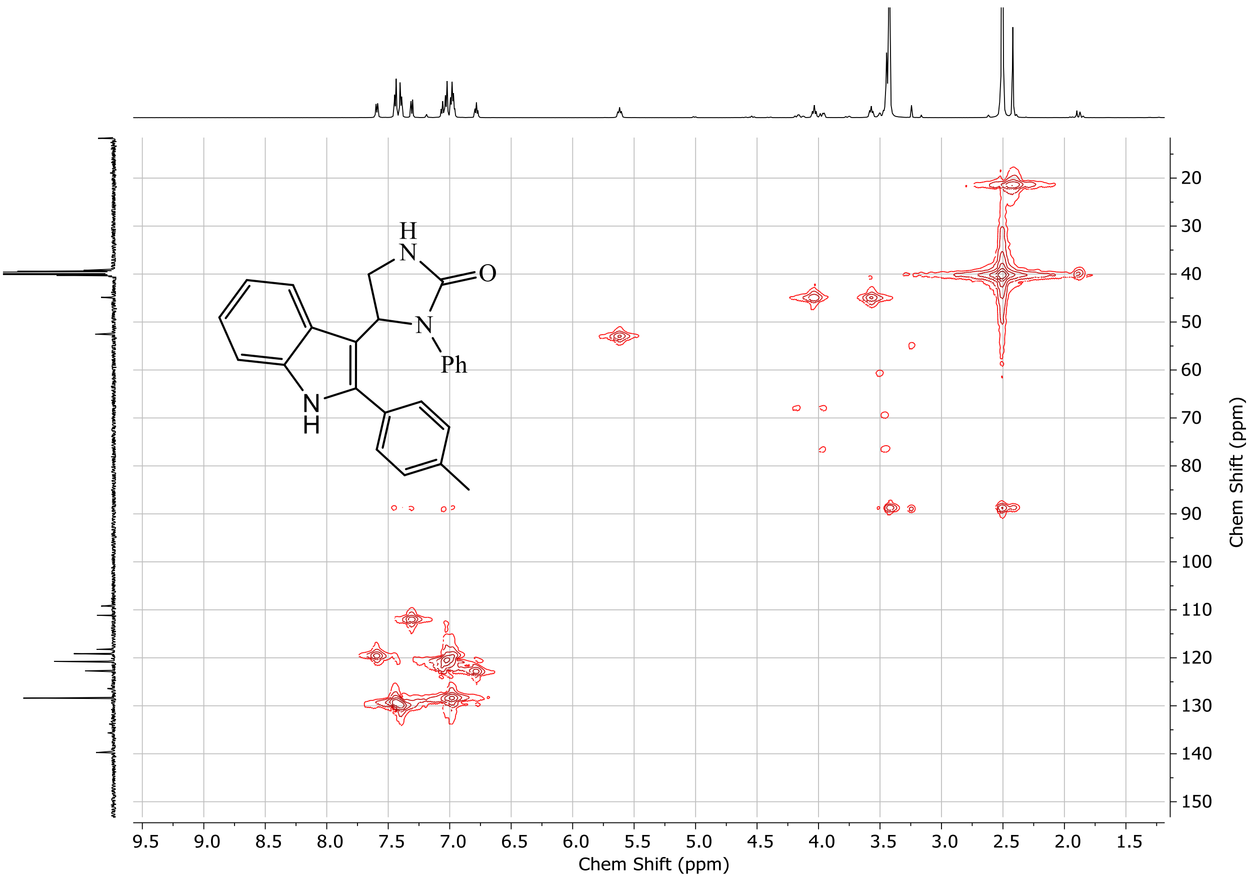

Figure S11. HMQOGR spectrum of 5-(2-p-Tolyl-1H-indol-3-yl)-1-phenylimidazolidin-2-one **3c**.

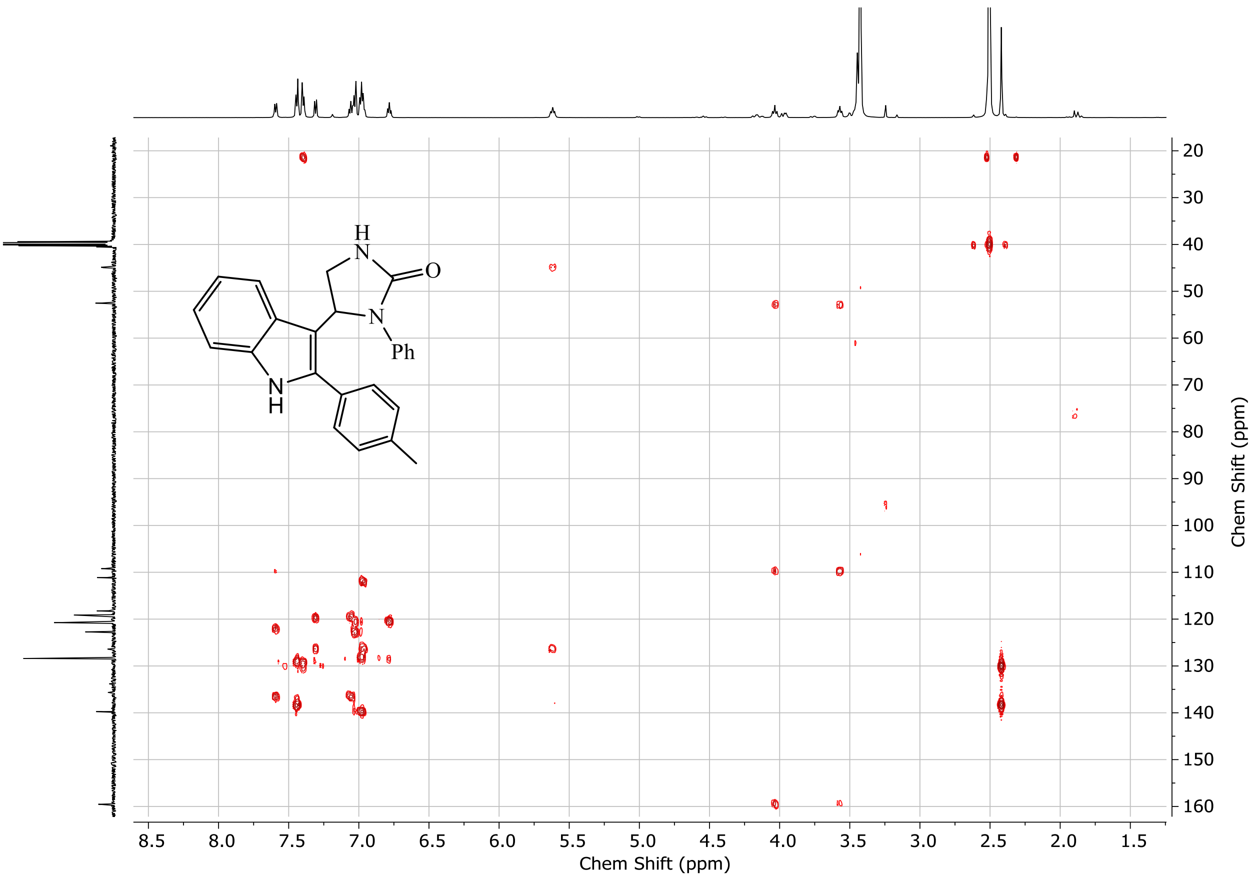

Figure S12. gHMBC NMR spectrum of 5-(2-*p*-tolyl-1*H*-indol-3-yl)-1-phenylimidazolidin-2-one **3c**.

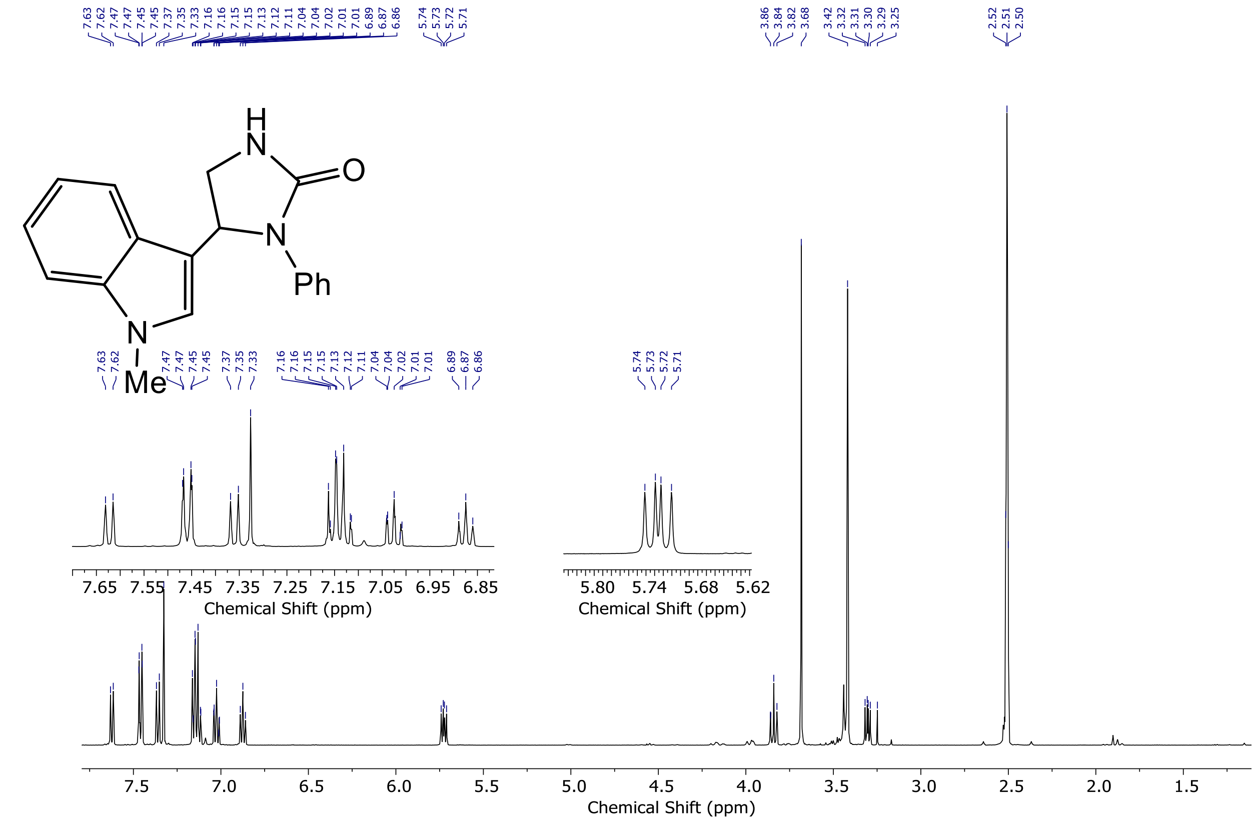

Figure S13. ^{1}H NMR spectrum of 5-(1-methy-1*H*-lindol-3-yl)-1-phenylimidazolidin-2-one **3d**.

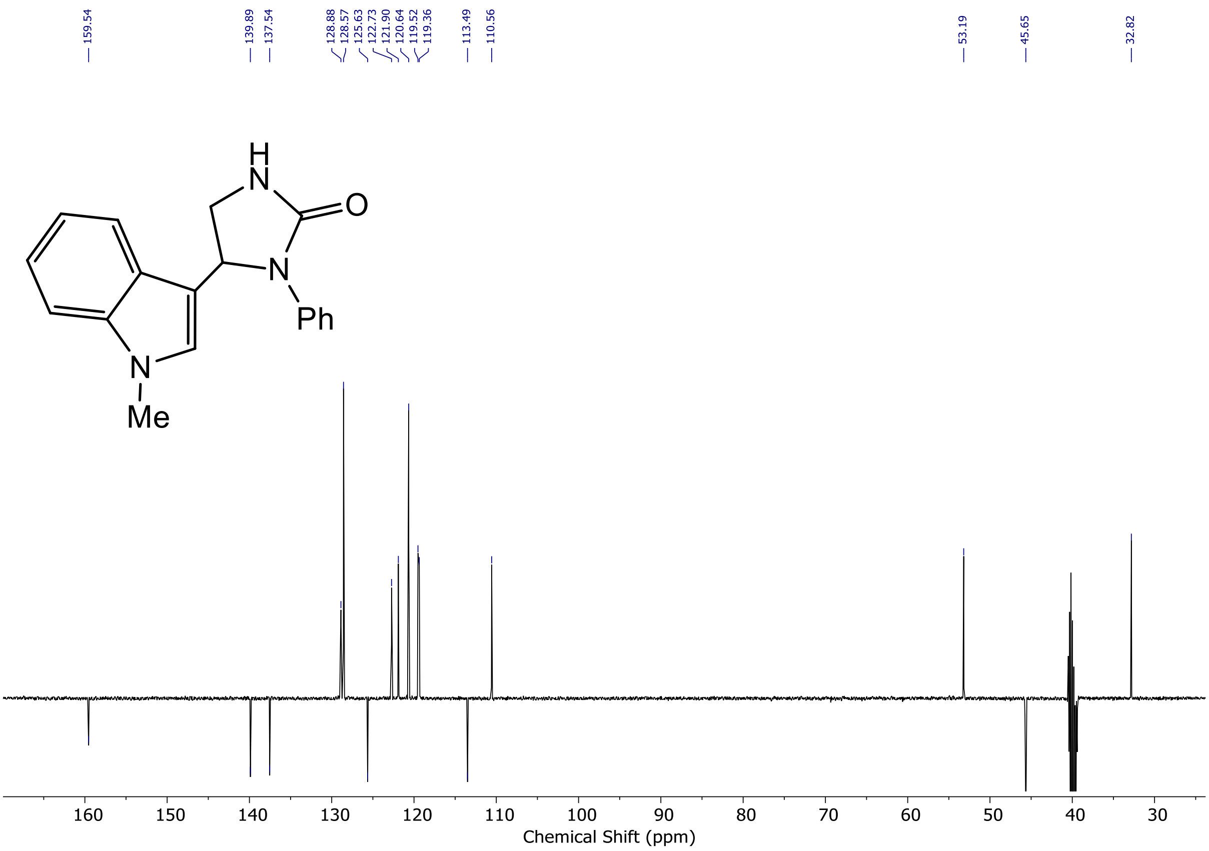

Figure S14. ^{13}C NMR spectrum of 5-(1-methy-1*H*-lindol-3-yl)-1-phenylimidazolidin-2-one **3d**.

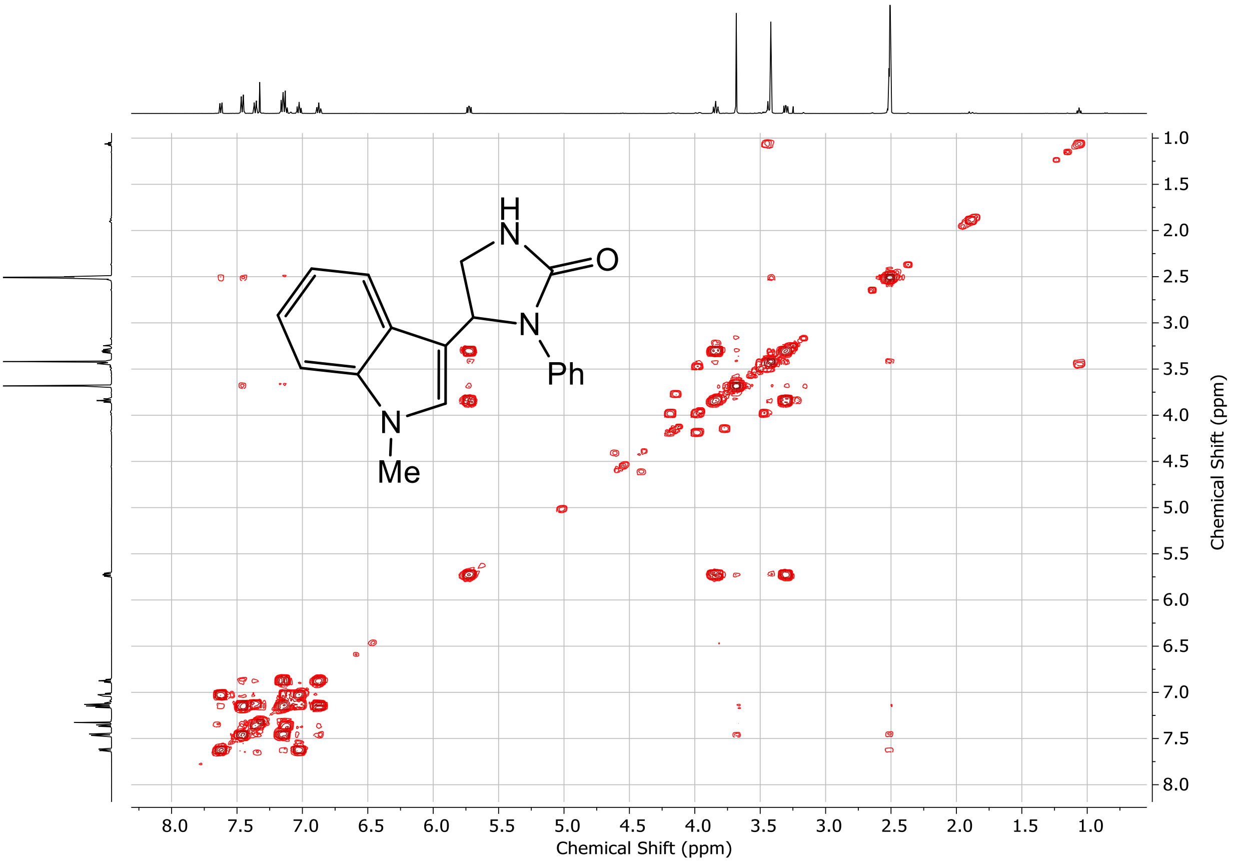

Figure S15. gCOSY NMR spectrum of 5-(1-methy-1*H*-lindol-3-yl)-1-phenylimidazolidin-2-one **3d**.

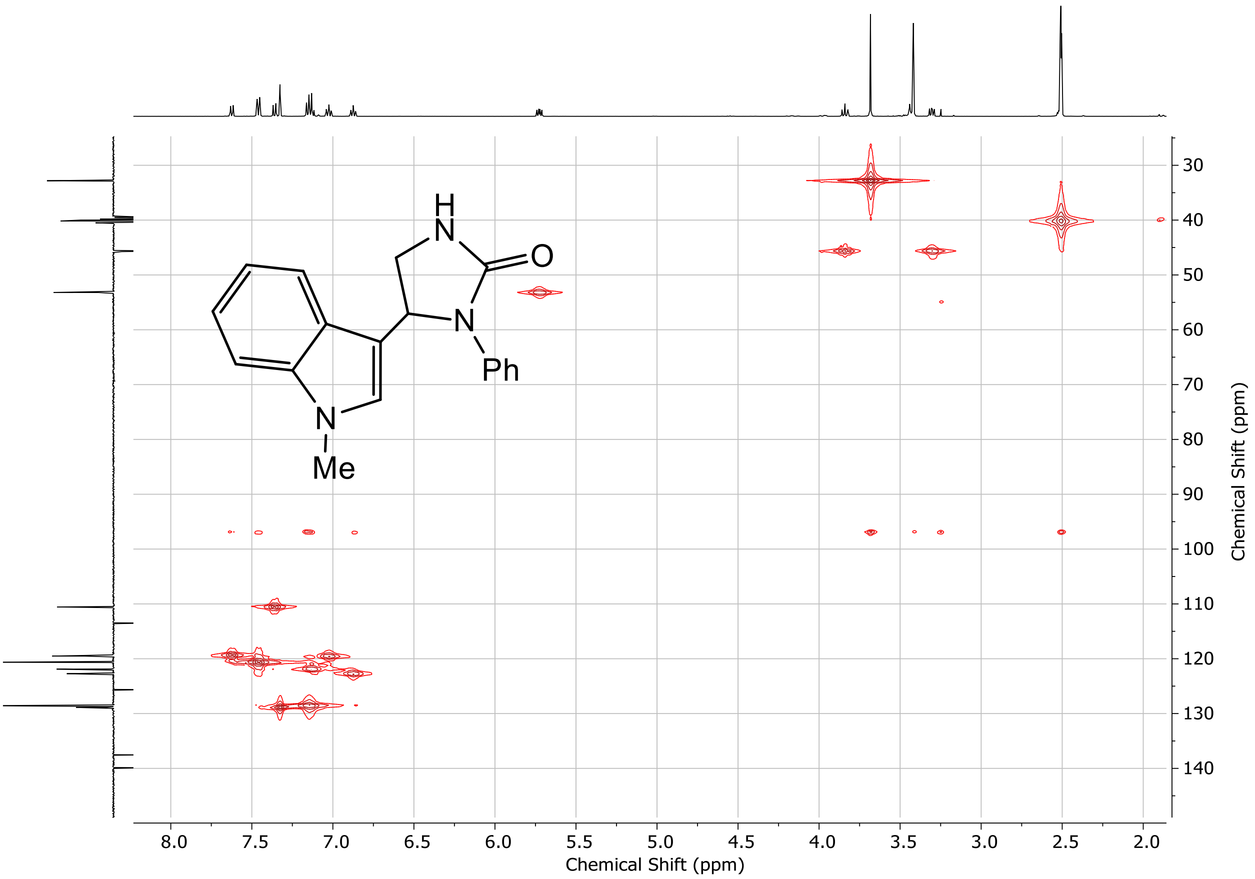

Figure S16. HMQOGR NMR spectrum of 5-(1-methy-1*H*-lindol-3-yl)-1-phenylimidazolidin-2-one **3d**.

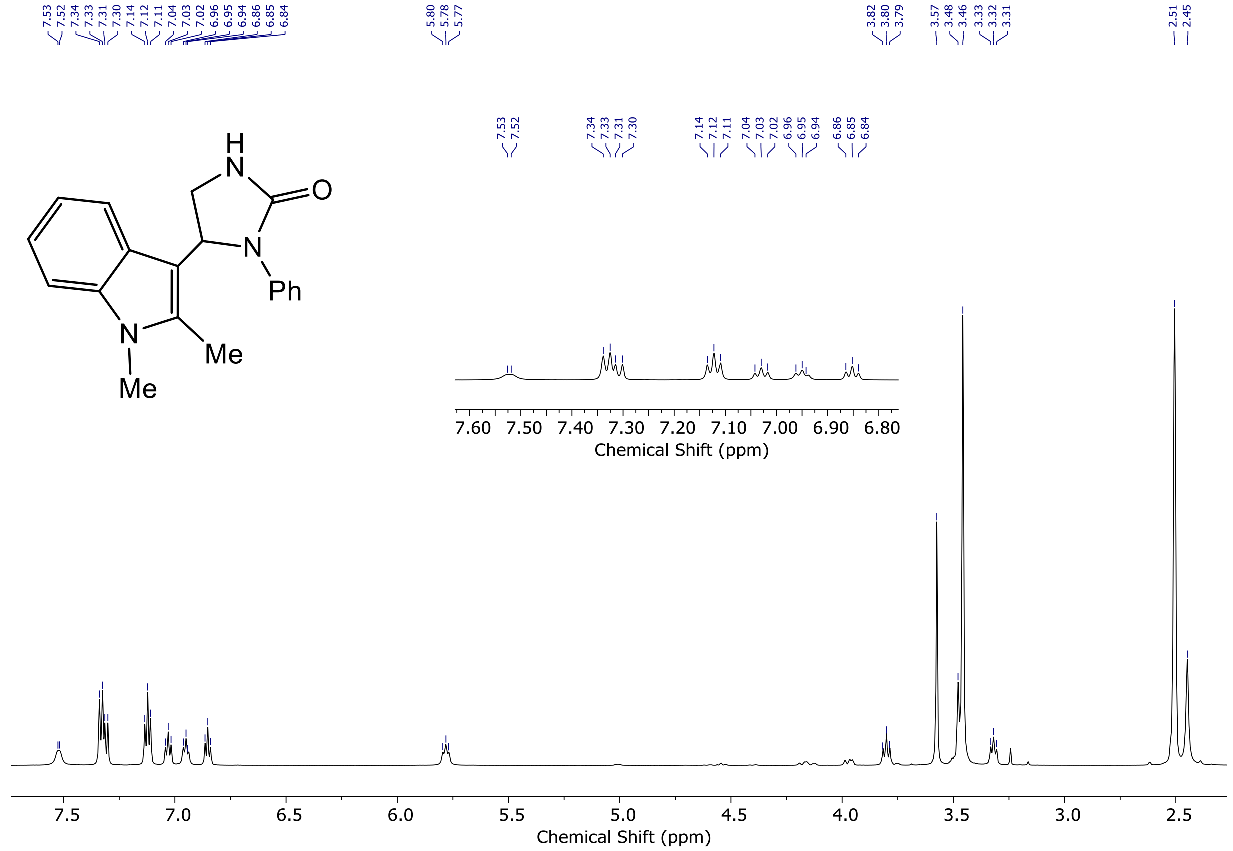

Figure S17. ^{1}H NMR spectrum of 5-(1,2-dimethyl-1*H*-indol-3-yl)-1-phenylimidazolidin-2-one **3e**.

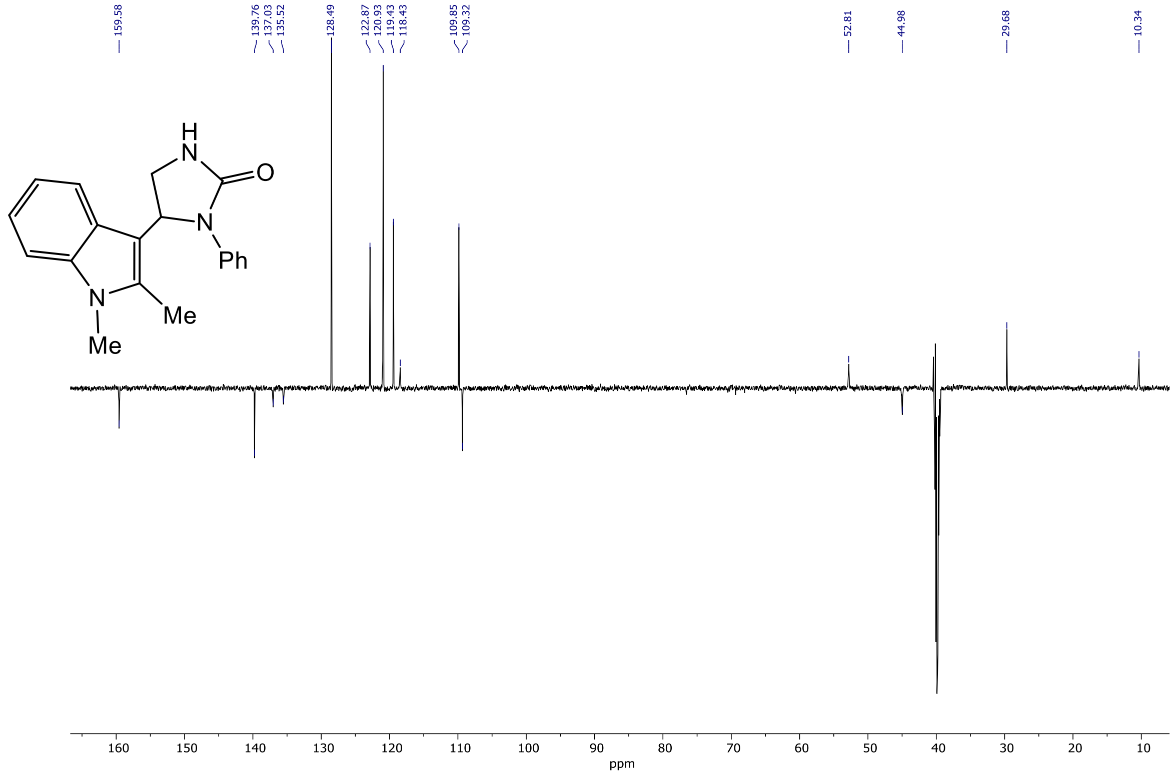

Figure S18. ^{13}C NMR spectrum of 5-(1,2-dimethyl-1*H*-indol-3-yl)-1-phenylimidazolidin-2-one **3e**.

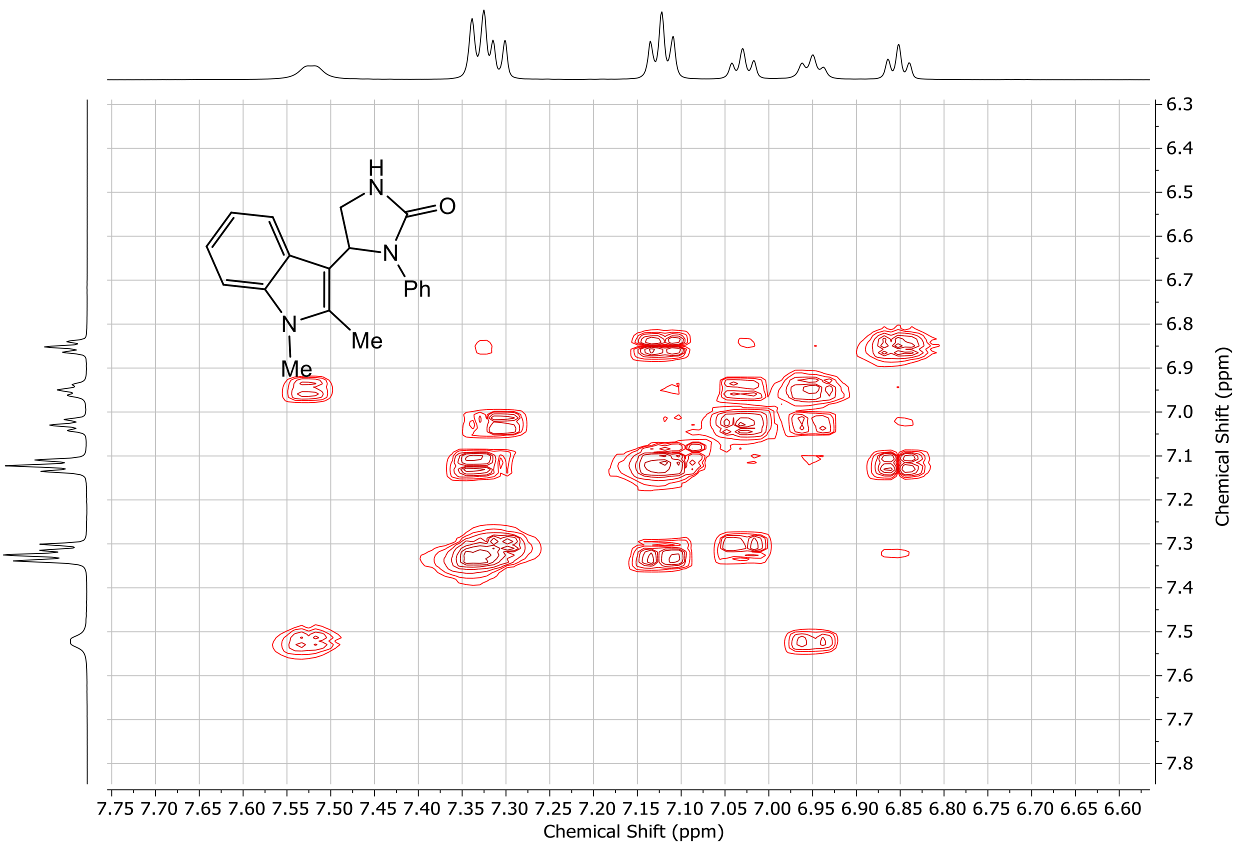

Figure S19. gCOSY NMR spectrum of 5-(1,2-dimethyl-1*H*-indol-3-yl)-1-phenylimidazolidin-2-one **3e**.

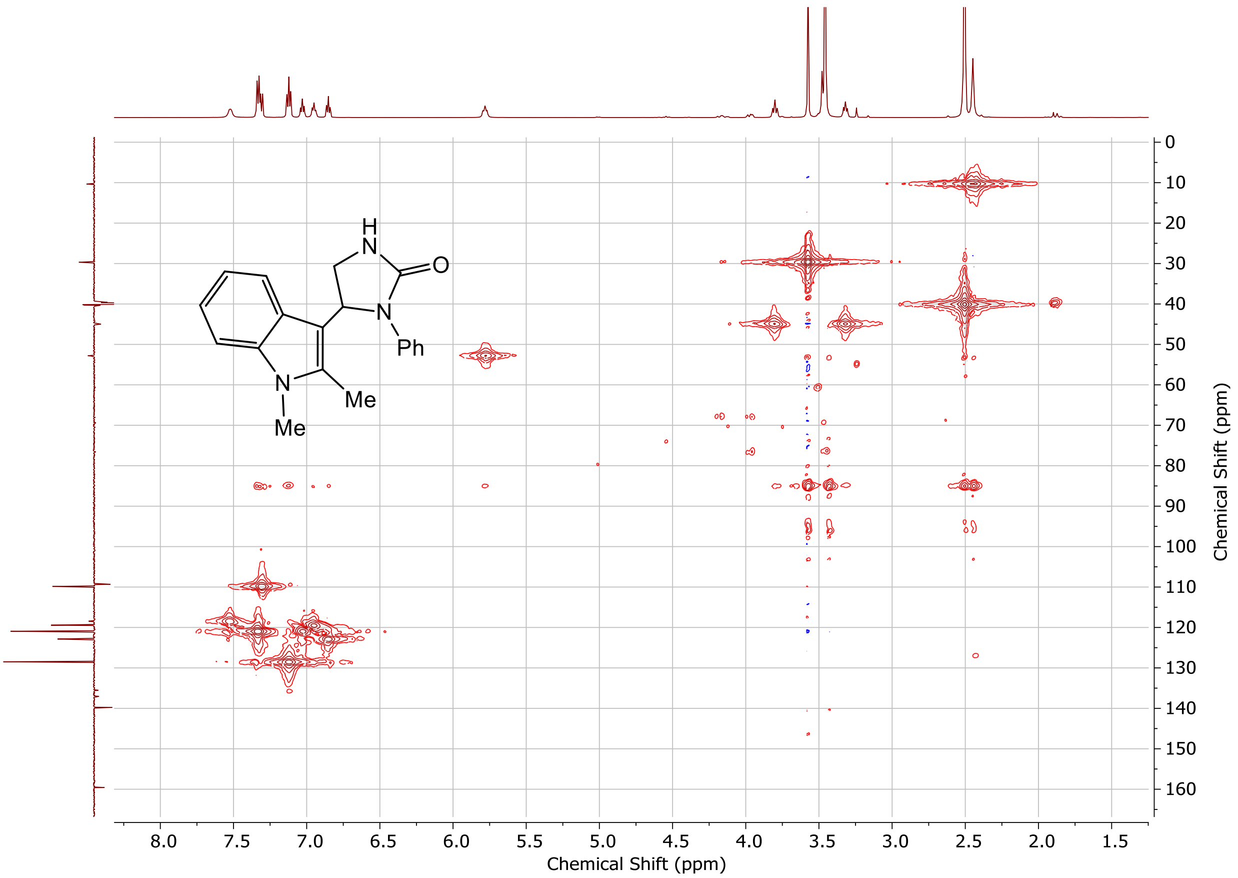

Figure S20. HMQOGR NMR spectrum of 5-(1,2-dimethyl-1*H*-indol-3-yl)-1-phenylimidazolidin-2-one **3e**.

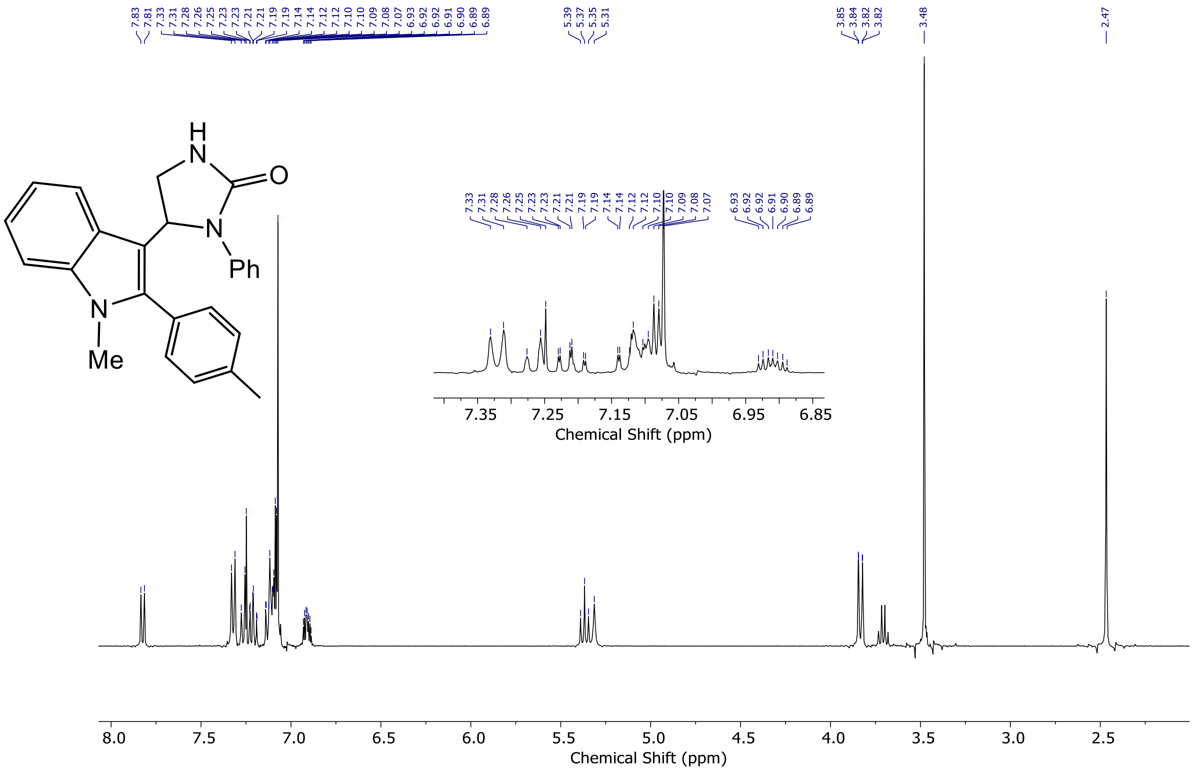

Figure S21. ^{1}H NMR spectrum of 5-(1-methyl-2-*p*-tolyl-1*H*-indol-3-yl)-1-phenylimidazolidin-2-one **3f**.

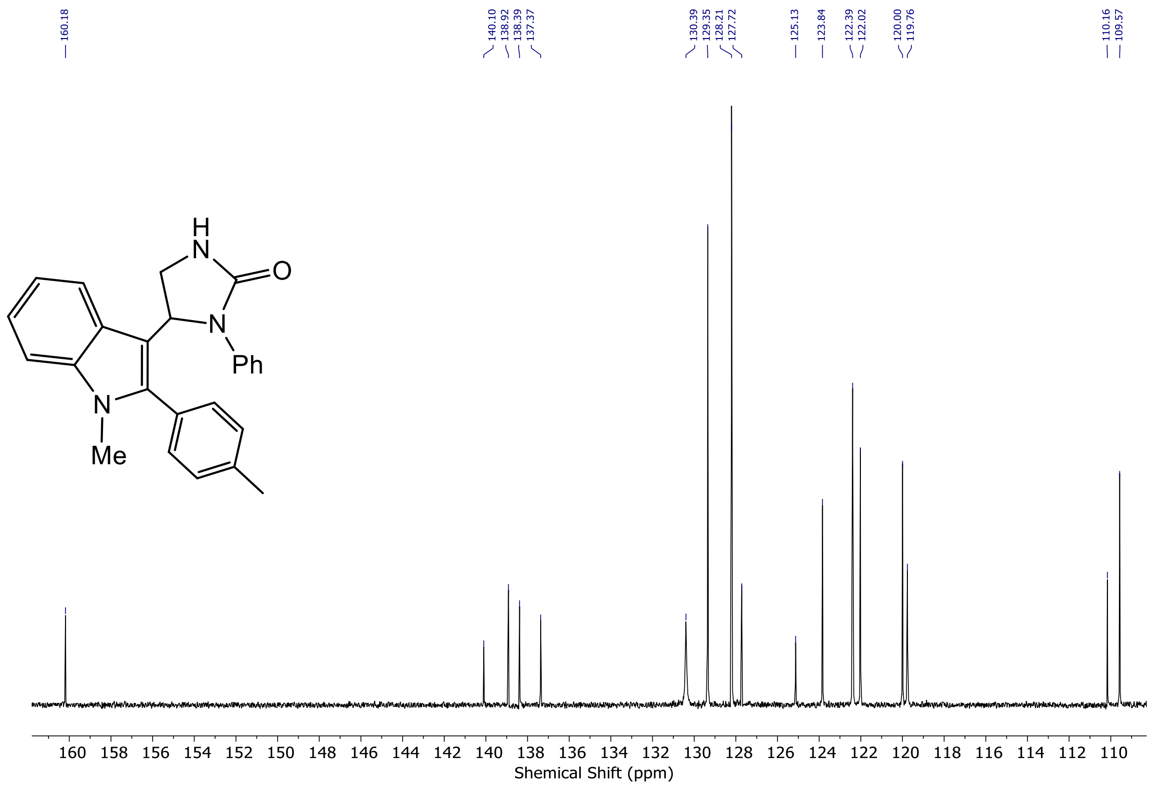

Figure S22. ^{13}C NMR spectrum of 5-(1-methyl-2-*p*-tolyl-1*H*-indol-3-yl)-1-phenylimidazolidin-2-one **3f**.

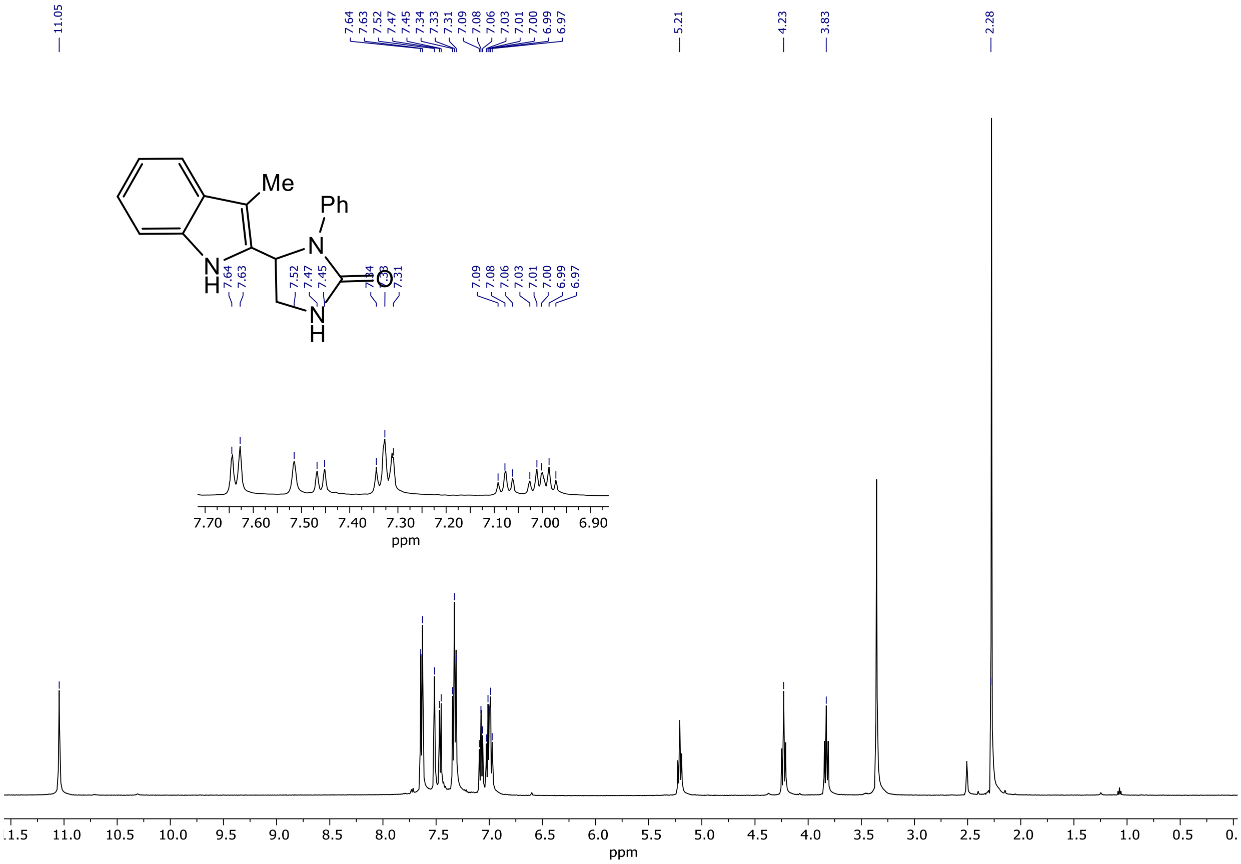

Figure S23. ^{1}H NMR spectrum of 5-(3-methyl-1*H*-indol-2-yl)-1-phenylimidazolidin-2-one **3g**.

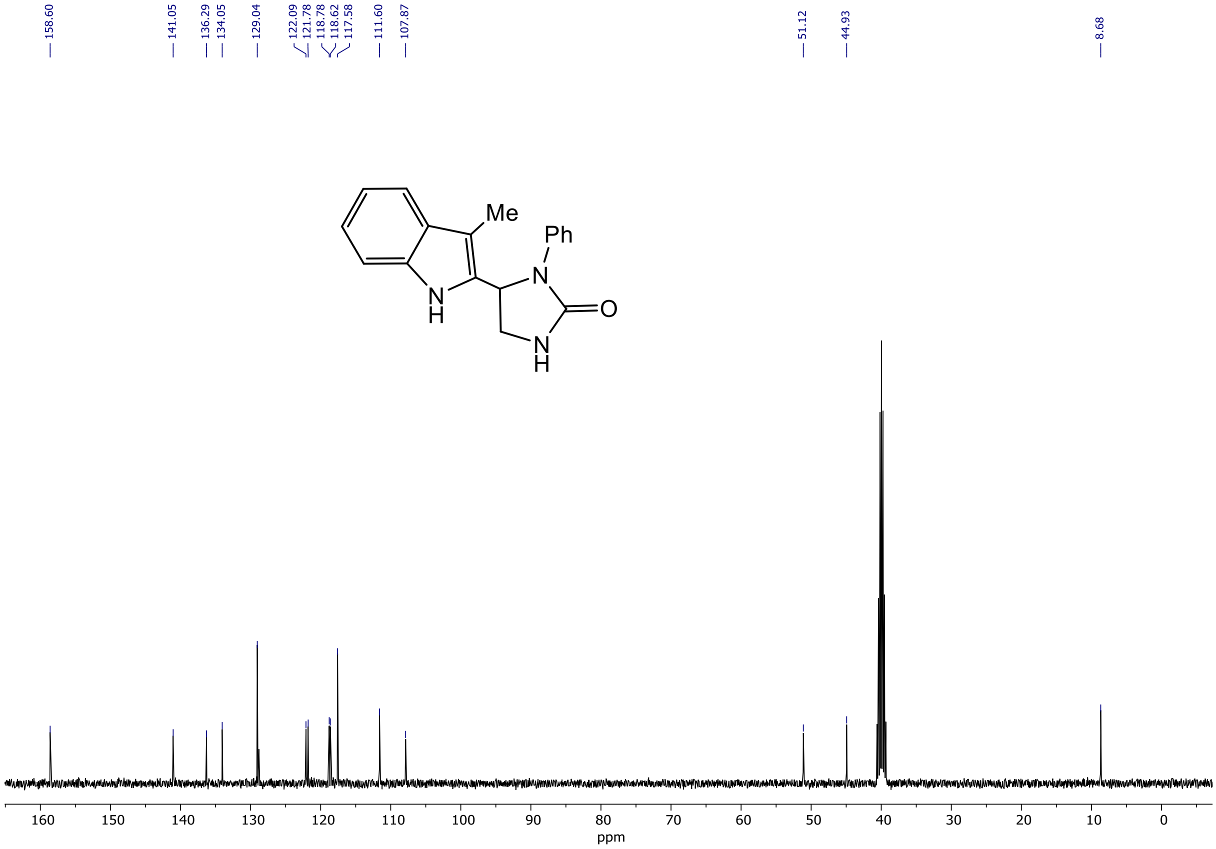

Figure S24. ^{13}C NMR spectrum of 5-(3-methyl-1*H*-indol-2-yl)-1-phenylimidazolidin-2-one **3g**.

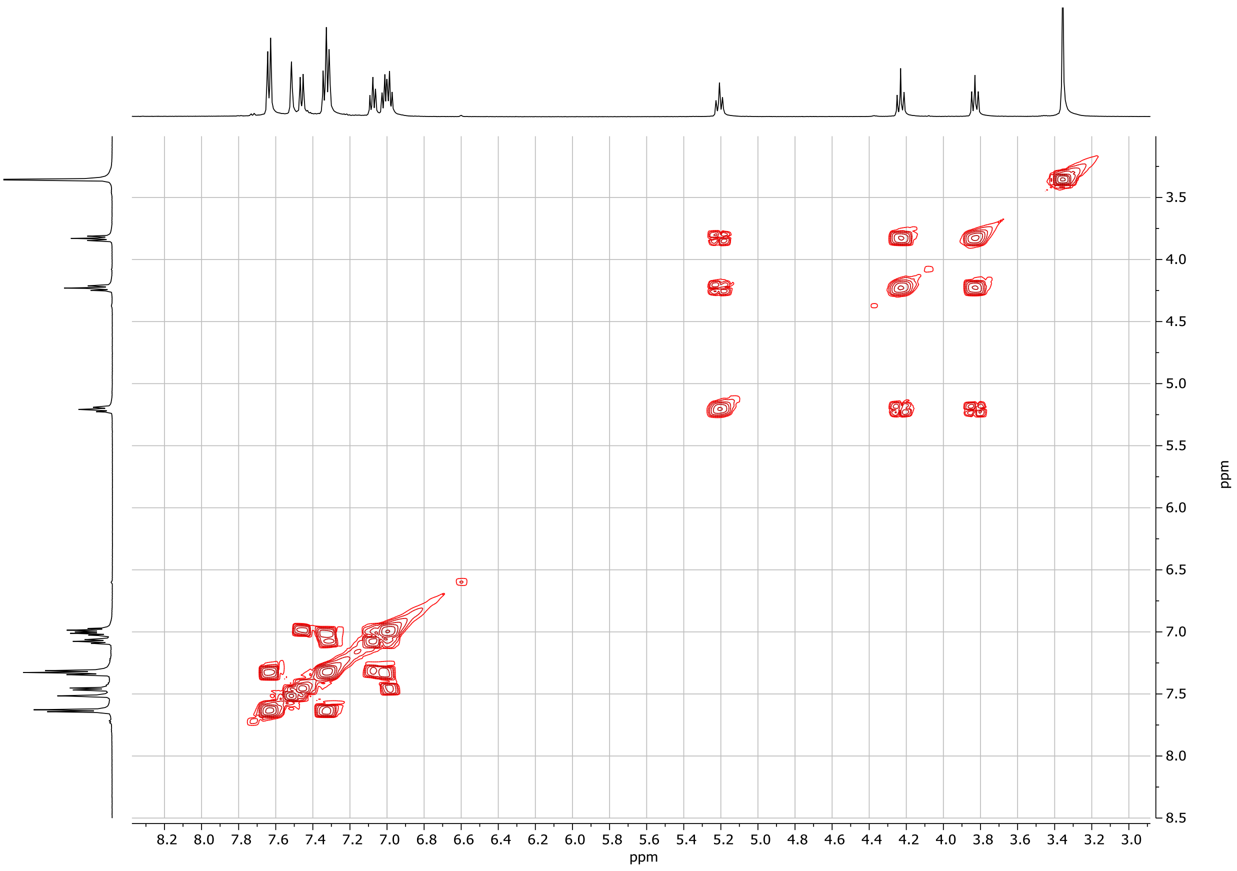

Figure S25. gCOSY spectrum of 5-(3-methyl-1*H*-indol-2-yl)-1-phenylimidazolidin-2-one
